# The effect of different flushing and locking techniques on catheter occlusion rates in central venous catheters: protocol for a multicentre, randomized controlled, parallel-group, open-label, superiority clinical trial

**DOI:** 10.1186/s13063-024-08141-6

**Published:** 2024-06-12

**Authors:** Rongmei Li, Mian Zhou, Lulu Sun, Lili Sha, Biyun Xu, Taishun Li, Tingting Tao, Ling Yuan

**Affiliations:** 1 Oncology Department, Nanjing Drum Tower Hospital, Affiliated Hospital of Medical School, Nanjing University, Nanjing, China; 2 Nanjing Drum Tower Hospital, Affiliated Hospital of Medical School, Nanjing University, Nanjing, China

**Keywords:** Central venous catheter, Intermittent flushing, Continuous infusion, KVO, Catheter patency

## Abstract

**Background:**

Maintaining venous access is of great clinical importance. Running a slow continuous infusion to keep the vein open (KVO) is often used in peripheral intravenous catheters (PIVCs). Previous studies have compared the effects of intermittent flushing and continuous infusion via peripherally inserted central catheters (PICCs). In this study, we applied KVO to central venous catheters (CVCs) and compared the occlusion rate of this technique with that of the intermittent flushing technique.

**Method:**

This is a randomized controlled trial of 14 hospitals in China. A total of 250 patients will be recruited in this study, and they will be randomized at a 1:1 ratio. After study inclusion, patients who will undergo CVC insertion will receive intermittent flushing with prefilled saline syringes (control group) or KVO infusion with elastic pumps (test group). All the catheters will be checked for patency by scoping Catheter Injection and Aspiration (CINAS) Classification on Days 3 and 7. The primary outcome is the rate of catheter occlusion in 7 days. Patients will be followed up until 9 days after CVC insertion, catheter occlusion, or catheter removal. The secondary outcomes are the rate of catheter occlusion in 3 days, nurse satisfaction, cost-effectiveness, adverse event rate, catheter-related bloodstream infection rate, catheter-related thrombosis rate, extravasation rate, phlebitis rate, and catheter migration.

**Discussion:**

We expect that the trial will generate findings that can provide an evidence-based basis for the improvement and optimization of clinical catheter flushing techniques.

**Trial registration:**

Chinese Clinical Trial Registry, ChiCTR2200064007. Registered on 23 September 2022. https://www.chictr.org.cn/showproj.html?proj=177311.

**Supplementary Information:**

The online version contains supplementary material available at 10.1186/s13063-024-08141-6.

## Administrative information

**Table:** 

Title **{1}**	The effect of different flushing and locking techniques on catheter occlusion rates in the central venous catheter (CVC): protocol for a multicentre, randomized controlled, parallel-group, open-label, superiority clinical trial
Trial registration **{2a and 2b}**	Chinese Clinical Trial Registry (ChiCTR, www.chictr.org.cn) No. ChiCTR2200064007. Registered on 23 September 2022. https://www.chictr.org.cn/showproj.html?proj=177311
Protocol version** {3}**	Protocol version 2, July 20, 2022
Funding** {4}**	Nanjing Drum Tower Hospital transverse research project (2022-417-02; Study of the effectiveness of different flushing and locking techniques for central venous catheter(CVC)care).
Author details **{5a}**	Nanjing Drum Tower Hospital, Affiliated Hospital of Medical School, Nanjing University, Nanjing, China
Name and contact information for the trial sponsor **{5b}**	Investigator-initiated clinical trial; Ling Yuan (Principal Investigator) yuanling@njglyy.com
Role of sponsor **{5c}**	The role of the sponsor: investigator.

## Introduction

### Background and rationale {6a}

Maintaining unobstructed venous access is of great clinical importance [1, 2]. The central venous catheter is widely used in clinical practice, with single-lumen, double-lumen, and multi-lumen, and can be used for monitoring hemodynamic, infusion therapy, blood transfusion, chemotherapy, and parenteral nutrition [3, 4]. The complications associated with central venous catheters can reach 40% [5], and common complications include occlusion, infection, and catheter dislodgement [6], with occlusion occurring in 25% to 38% of cases [7]. Blockage of a catheter can affect the patient’s treatment [8] or, worse, can threaten the patient’s life and increase health care costs [9].

The central venous catheter occlusion factors can be divided into intrinsic and extrinsic factors. Intrinsic factors, such as advanced age, female sex, hypercoagulable blood, and disease, are related to the patients themselves and can lead to an increased probability of catheter occlusion [10]. Extrinsic factors include the catheter placement site, number of punctures, catheter folding, improper flushing, and nature of the infused drug [11, 12]. Improper flushing, for example, failure to flush the lumen in a timely manner, failure to use the correct locking solution, or failure to use the correct flushing technique, can increase the probability of catheter occlusion [13, 14]. In addition, infusion of specific drugs can also lead to occlusion; for example, infusion of drugs that are hypertonic, too high, or too low in pH can easily lead to changes in plasma osmolality, thrombus formation, catheter tip occlusion, or haemagglutination occlusion [15].

Keep vein open (KVO) refers to the practice of keeping a vein open with a small amount of intravenous solution [16]. Previous studies have focused mainly on comparing the effects of intermittent flushing and continuous infusion on peripheral intravenous catheters and neonates [17, 18]. However, none of these studies have offered a comparison in the adult population. In this study, we will ameliorate KVO infusion and use it in the adult population. We will use an elastic pump to run continuous infusion rather than gravity infusion. This kind of elastic pump contains 50 ml of 0.9% sodium chloride solution, and KVO infusion can be applied at a rate of 2 ml/h. KVO infusion was used between doses of intermittent medication in previous studies. In this study, participants will receive a whole-day KVO infusion, regardless of whether the participants are on intermittent medication.

Studies [19–21] have shown that the use of an elastic pump achieves the same results as heparin locking in general surgery patients with discontinuous infusions using single-lumen central venous catheters and in the maintenance of peripheral venous catheters, ensuring 100% catheter patency. The elastic pump infusion technique is designed to maintain the infusion pressure at 55 kPa, which is much higher than the human central venous pressure (0.5 ~ 1.2 kPa), by adopting the clinical micropump and infusion pump precision infusion principal technique and the built-in flow limiter, which can achieve continuous dynamic infusion and maintain catheter patency.

It is worthwhile to further investigate what kind of flushing technique can better ensure the patency of anterior-open multilumen central venous catheters (CVCs), which are widely used in continuous infusions, to ensure the safety of patient treatment and ease of movement. We completed a pretest with a sample of 20 patients to test the effectiveness of the KVO flushing technique for maintaining the patency of dual-lumen CVCs. The experimental results showed that no catheter occlusion occurred in the test group, while two cases of catheter occlusion occurred in the control group. Therefore, our team plans to expand the sample size and design a multicentre clinical control trial to test the effectiveness of this technique.

### Objectives {7}

The primary objective is to determine whether flushing a catheter under KVO infusion is superior to flushing a catheter under prefilled saline syringes. The secondary objectives are to establish and optimize the flushing and locking technique for CVC and establish standardized operating procedures (SOPs) for this technique.

### Trial design {8}

This is a multicentre**,** randomized controlled, parallel-group, open-label, superiority clinical study with two groups: (1) patients in the test group will receive KVO infusion to maintain CVC patency with elastic pumps (GEMTIER MEDICAL, 50 ml, 2 ml/h) and (2) patients in the control group will receive routine care.

**Fig. 1:**
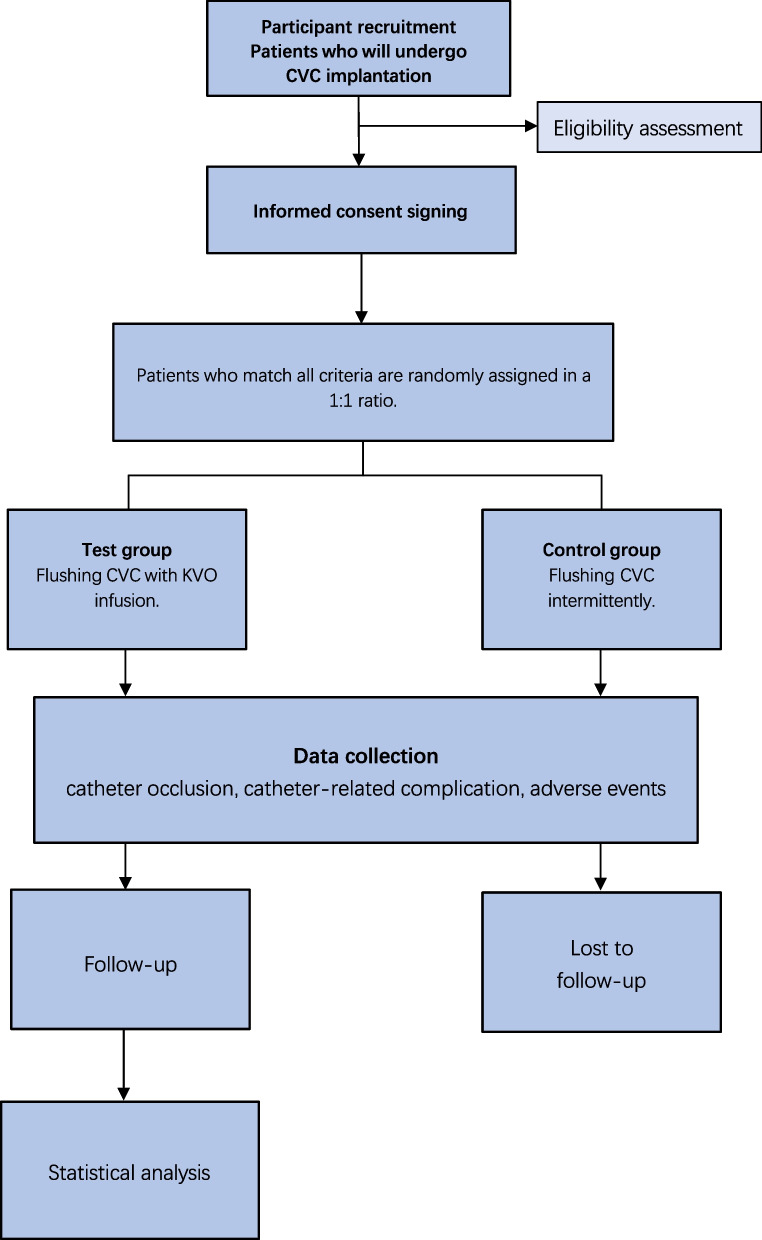
Flowchart of the study

After providing informed consent from patients, they will be assigned to either the test group (with continuous flushing of the CVC under KVO infusion) or the control group (with intermittent flushing of the CVC with prefilled saline syringes) at a 1:1 ratio (Fig. 1).

## Methods: Participants, interventions, and outcomes

### Study setting {9}

The 14 centres included in this study are located in Jiangsu Province, Anhui Province, and Hunan Province, China. The departments involved in this study include the intensive care unit (ICU), surgical ward, and oncology ward.

#### Eligibility criteria {10}

The inclusion criteria for patients will be as follows: (1) hospitalized using an anterior-opening double-lumen CVC; (2) aged 14 to 80 years; (3) newly placed CVC within 24 h; (4) had a normal catheter function (catheter injection and aspiration CINAS judged as IN1AS1); (5) voluntarily participated in this research; (6) had CVC placed through the internal jugular vein or subclavian vein; and (7) had a daily continuous infusion time ≥ 16 h. The exclusion criteria for patients will be as follows: (1) had existing catheter-related complications; (2) uses drugs incompatible with 0.9% sodium chloride solution; (3) uses drugs that cannot tolerate stopping infusion for flushing and locking the catheter; and (4) were locked with other locking solutions.

#### Who will take informed consent? {26a}

Informed consent will be obtained from the sub-investigators (SIs). During the trial screening period, the SIs will be responsible for subject screening. Patients in the ward who are possibly eligible for enrolment will be selected and informed about this trial in detail. If the patient agrees to participate in the study, then the patient will need to sign an informed consent form.

#### Additional consent provisions for collection and use of participant data and biological specimens {26b}

This trial does not involve collecting biological specimens for storage.

### Interventions

#### Explanation for the choice of comparators {6b}

The INS guidelines recommend the use of the pulsatile flushing technique [22]. The pulsatile flushing technique is widely used in clinical practice, and choosing this technique as a control group can improve the detection efficacy of the test group technique.

#### Intervention description {11a}

The placement and maintenance of CVCs are carried out by trained and qualified professionals. Disinfectants that meet Chinese regulations [23] are selected for puncture and maintenance, creating a maximum sterile barrier and using a uniform catheter placement process. We previously standardized the type of CVC (FORNIA, CVC-2 7F 20) to be applied in this study to reduce interference. In addition to the flushing technique, other care measures for the catheter will be carried out according to the same standard, including using the same infusion connector (BD Luer-Lok™ 394605), changing the dressings once a week**,** and changing the infusion once a day; in special cases, the frequency of replacement will increase.

Depending on the grouping, patients in the test group will undergo KVO infusion using an elastic pump containing 50 ml of 0.9% sodium chloride solution, which maintains a constant pressure (55 kPa) and a steady infusion rate (2 ml/h). Before daily infusion, the study nurses will mechanically wipe and disinfect the infusion connector, connect the stopcocks according to the infusion demand, connect the elastic pump to the female opposite port of the endmost stopcocks, connect the medication pathways to the female side ports, adjust the stopcocks handle for infusion and flushing, and fix the elastic pump appropriately to observe the liquid in the elastic pump and pumping situation at regular intervals. At the end of the infusion, the stopcock handle will be adjusted to close the medication pathway, and the pumping of the elastic pump will be continued. The study nurses will change the pumps once a day. The elastic pump pumping valve will be closed, the elastic pumps will be separated from the stopcocks or catheters, and the stopcocks will be mechanically disinfected. Two new elastic pumps will then be connected, the handle will be adjusted to continue pumping, and the elastic pumps will be properly fixed to ensure that it does not interfere with patient activities.

In the control group, prefilled saline syringes containing 10 ml of 0.9% sodium chloride injection will be used to flush and lock the catheter via pulsatile flushing and the positive pressure locking technique. Before each infusion, the study nurses will mechanically wipe and sterilize the infusion connector. Catheter function will be assessed by flushing the catheters and aspirating for blood return. After seeing the blood return, the catheter will be flushed using short-interval pulsatile flushing (1 ml each time), for a total of 10 ml [22]. At the end of the infusion, prefilled saline syringes will be used to flush each lumen (even if only one lumen is used) via pulsatile flushing and the positive pressure technique [24].

The study will continue for 7 days unless there is catheter occlusion or catheter removal. During the 7 days, the study nurses will identify catheter occlusion by scoping the CINAS, observing the infusion status, and identifying the occlusion alarm. Once catheter occlusion occurs, the patients reach the end of the study, and the management of the blocked catheter will be referred to the physician and nurse. The end of the trial also includes a 2-day observation period, which will monitor the occurrence of catheter-related bloodstream infection (CRBSI), primarily through temperature monitoring.

Throughout the trial, the study team will fully respect the patient’s wishes, and the patients will have the right to withdraw from the trial. In addition, if a patient has no observed occlusion during the trial period and removes the CVC for other reasons, then the patient will be considered to have dropped out.

#### Criteria for discontinuing or modifying allocated interventions {11b}

The intervention will stop in the following circumstances:

1. Patients who developed planned or unplanned catheter removal;
2. Discharge, death, deterioration of the condition;
3. Patients withdraw proactively;
4. Wrong intervention.

#### Strategies to improve adherence to interventions {11c}

Before the start of the study, each centre will designate several nurses to perform the intervention. These nurses are competent in clinical practice. To exclude the influence of operational error on the study results, the leading unit conducted training to ensure that these nurses are familiar with the study. In addition, the leading unit has shot videos to standardize each operator’s practice. During the study, the leading unit will send coordinators to other centres to check protocol implementation, the most important of which is whether the intervention is regulated.

#### Relevant concomitant care permitted or prohibited during the trial {11d}

This is a randomized controlled study, and contamination between the two groups should be avoided. Therefore, pulsative flushing is prohibited for the test group.

#### Provisions for post-trial care {30}

After the trial, participants in both groups will resume routine care. There are no expected injuries or compensation for participation in the trial.

#### Outcomes {12}

The primary outcome of this study is occlusion rate in 7 days. Catheter occlusion will be assessed by scoping CINAS. The CINAS [25] is an instrument used to assess catheter function. It has two dimensions: injection ability (IN) and aspiration ability (AS). Each dimension includes four classifications: (1) easy, (2) difficult, (3) impossible, and (4) unknown. The CINAS comprises 16 possible ways to combine four distinct codes (Table 1). Each combination represents the ability to inject at least 1 ml of fluid and to aspirate at least 3 ml of blood and is graded on a scale of 1 to 3 (1 being easy, 2 being difficult, and 3 being impossible). When the injection and/or aspiration ability is unknown, a fourth classification option is provided (X).

**Table 1:**
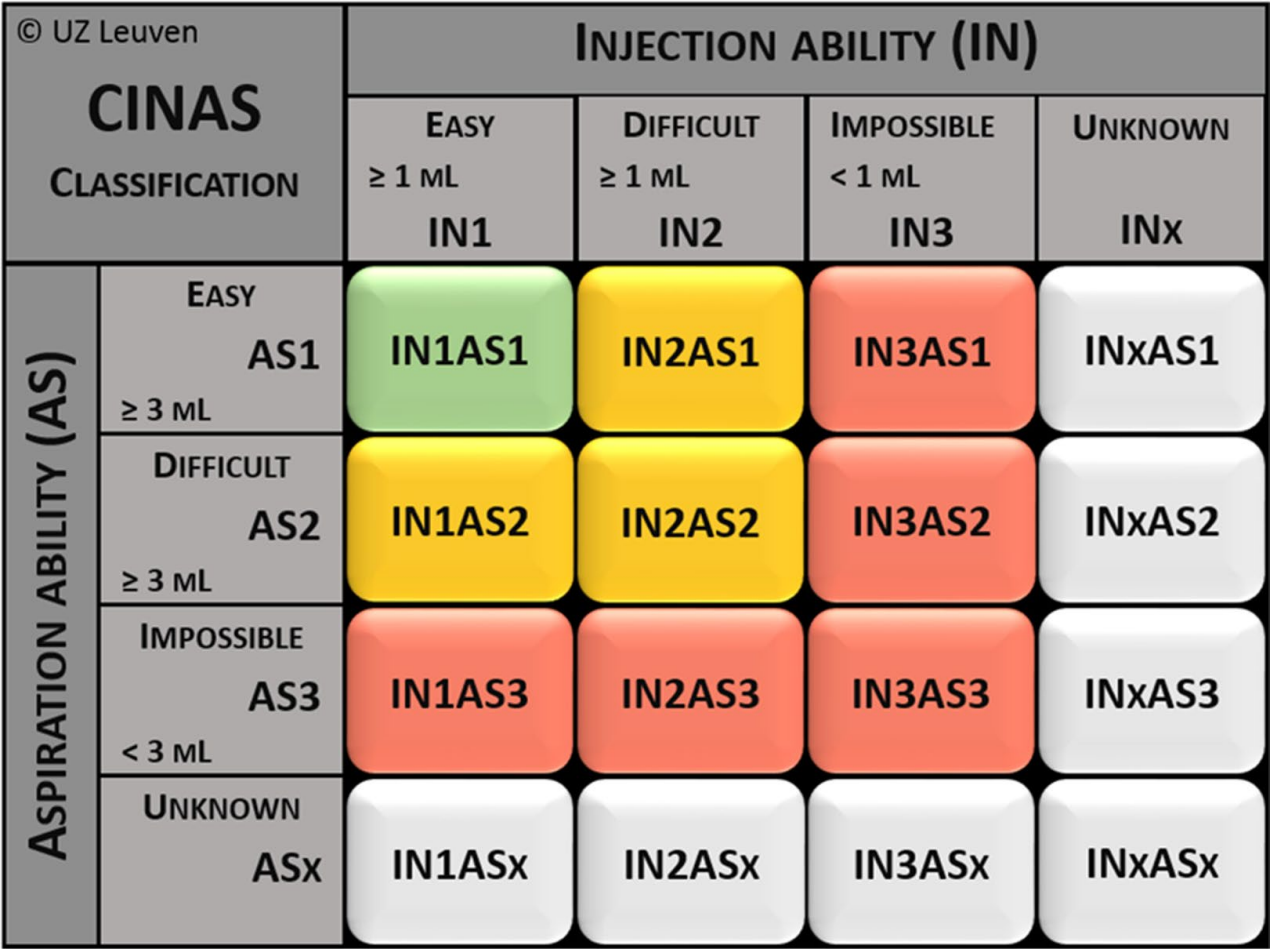
The Catheter Injection and Aspiration (CINAS) [25]

The secondary outcomes of this study include the 3-day catheter occlusion rate, which is also assessed by scoping CINAS, nurse satisfaction, and cost-effectiveness. Nurse satisfaction is defined as nurses’ attitudes towards these two different methods of flushing catheters. This outcome will be assessed by scoping a 5-point Likert scale (1 = Very dissatisfied, 2 = Dissatisfied, 3 = Unsure, 4 = Satisfied, 5 = Very satisfied). Cost-effectiveness will be assessed by calculating related medical expenses and nursing time and analysing the effect of the two methods.

The safety outcomes of this study are adverse event rates and intravenous therapy-related complications. The adverse events include unplanned removal, unplanned thrombolysis, and elastic pump failure. All these adverse events are defined as follows:

(I) Unplanned removal: intubation accidental dislodgement or removal of the catheter by the patient without the consent of the health care provider. This removal may also be the result of improper handling by the health care provider.
(II) Unplanned thrombolysis: a thrombotic occlusion of the patient’s CVC and the CVC needs to be recanalized with a thrombolytic drug such as a fibrinogen activator.
(III) Elastic pump failure: elastic pump breakage.

Intravenous therapy-related complications include CRBSI, catheter-related thrombosis (CRT), catheter misplacement, extravasation, and phlebitis. The definitions of these metrics are shown below:

(I) CRBSI: bacteraemia or fungemia in a patient with an intravascular catheter or within 48 h of removal of an intravascular catheter with manifestations of infection such as fever (38 °C), chills, or hypotension, with no clear source of infection other than the vascular catheter. Laboratory microbiology reveals positive peripheral venous blood cultures for bacteria or fungi or pathogenic bacteria of the same species with the same drug-sensitive results from the catheter segment and peripheral blood cultures.
(II) CRT: a mural thrombus extending from the catheter into the lumen of a vessel, leading to partial or total catheter occlusion with clinical symptoms that was established by Doppler colour flow imaging.
(III) Catheter misplacement: the position of the catheter tip changes.
(IV) Extravasation: fluid or drug extravasation.
(V) Phlebitis: this includes chemical phlebitis, mechanical phlebitis, and infectious phlebitis. Clinical signs and symptoms include pain, pressure, erythema, and erythema. Grades will be assessed according to the phlebitis scale in the INS guidelines [22].

#### Participant timeline {13}

As shown in Table 2.

**Table 2:** Participant timeline {13}

Study period			
Timepoint	Pre-study screening/consent	Study	Follow-up
	−1~0	1~7	8~9
Eligibility screen	×		
Informed consent	×		
Allocation	×		
Demographic information	×		
Medical history and treatment history	×		
Inclusion/exclusion form	×		
Vital signs	×	×	×
Mechanical ventilation		×	
Haematology(RBC/WBC/PLT/HBG)	×		
Coagulation(PT/APTT/FIB/D-Dimer/INR)	×		
Observe the catheter scale	×	×	
Intervention		×	
Assessing catheter patency	×	×	
Complication records	×	×	×
Adverse event		×	×
Medication record		×	
Catheter removal record		×	
Consumables		×	
Operation time		×	
Research summary			×

#### Sample size {14}

The primary outcome is the rate of catheter occlusion within 7 days. A preliminary study with a small number of patients (20 patients) indicated that the catheter occlusion rate was 0 in the test group and 20% in the control group. According to an expert consensus [7], the occlusion rate in the control group ranged from 25 to 38%. Using an estimation procedure based on the differences between the two groups, the necessary sample size was determined. An occlusion rate of 10% was assumed in the test group, and an occlusion rate of 25% was assumed in the control group. A minimum sample size of 125 participants per group (250 participants total) was calculated by PASS 15 software (NCSS, LLC) to provide sufficient statistical power for detecting the difference between the two groups. This was done by setting the single-sided significance level (*α*) at 0.025 and the statistical power (1-*β*) at 0.8 while taking into consideration a dropout rate of 20%.

The sample size was calculated as follows:

$${n}_{T} = {n}_{C}=\frac{{({Z}_{1-a/2}+ {Z}_{1-\beta })}^{2} \left[{P}_{C}\left(1-{P}_{C}\right)+{P}_{T}(1-{P}_{T}) \right]}{{\left(\left|D\right|-\Delta \right)}^{2}}$$

#### Recruitment {15}

Patient recruitment will be conducted at 14 medical centres in 3 provinces, Jiangsu, Anhui, and Hunan provinces, in China, and recruitment will be carried out for critically ill patients using CVC. Recruitment began in February 2023, and the study is scheduled to be completed within 3 months. Each centre plans to enrol 18 patients, which means that each centre will enrol 6 patients per month.

### Assignment of interventions: allocation

#### Sequence generation {16a}

Before the start of the study, the statistics team will perform randomization using a computer-generated random assignment list at a 1:1 ratio.

#### Concealment mechanism {16b}

The statistics team will generate random numbers and will hand them over to people unrelated to the study, who will then make sealed, opaque envelopes, and send them to each centre in order of the centres’ initials. Each centre will receive 18 envelopes. The random envelopes will be kept by the principal investigators (PIs) of each centre and will not be accessible to persons unrelated to this study. The statistics team will not be involved in clinical trials.

#### Implementation {16c}

The SIs are responsible for enrolling the subjects. After the patient signs the informed consent form, the SIs will assess the patient's eligibility for enrolment. For patients eligible for enrolment, the random envelope will be opened in the order of enrolment to obtain a unique random number.

### Assignment of interventions: blinding

#### Who will be blinded {17a}

It is difficult to blind participants and study investigators in this study, but the data analysts will be blinded. Data analysts will not participate in the clinical trial process, and they will not know the interventions used in the two groups.

#### Procedure for unblinding if needed {17b}

The design is open label, so unblinding will not occur.

### Data collection and management

#### Plans for assessment and collection of outcomes {18a}

We held 13 online training sessions to conduct case report form (CRF) completing, trial procure, participant assessment, and other relevant details. Before the trial begins, every centre will receive on-site instruction, and they will be encouraged to complete a pretest to ensure familiarity with the process. All the data will be entered into an electronic database by two trained researchers.

#### Plans to promote participant retention and complete follow-up {18b}

The objective of the study is to compare the effects of two flushing techniques over 7 days. To minimize the dropout rate, patients with a CVC usage duration of more than 8 days must be selected during the screening process. Given that the study cannot intentionally prolong the patient’s catheter-wearing time, the challenging task of selecting eligible patients is entrusted to the head nurses, who are well versed in clinical work. For those patients who are transferred within the hospital and have an intervention time of less than 7 days, the study will continue to track them unless they are discharged or transferred to another hospital.

#### Data management {19}

Statistical analysis and management of the data will be performed by a third-party team independent of the study. Data entry will be performed by two SIs specializing in the task, and the data will be entered once by each SI. Only when the two sets of data entered are checked without discrepancy can analysis be conducted.

All the statistical analysis will be performed with SAS 9.4 (SAS Institute Inc., Cary. NC) or above. Quantitative information will be described using the number of patients, mean, standard deviation, median, upper quartile, lower quartile, minimum, and maximum values. The categorical data will be described by the number of patients and percentage of each category. Comparisons between two general groups will be analysed using appropriate methods depending on the type of indicator. Group comparisons of quantitative data will be made using a group *t* test (chi-square test, normal distribution) or Wilcoxon rank sum test (if *t* test is not applicable) depending on the data distribution; chi-square test or Fisher's exact test (if chi-square test is not applicable) for categorical data; and the rank sum test for rank data. All the statistical tests will be performed using two-sided tests, and *p* values less than or equal to 0.05 will be considered to indicate statistical significance.

#### Confidentiality {27}

Each participant will be given a code upon enrolment. The statistics team will only see patient codes, and real information will not be disclosed.

### Plans for collection, laboratory evaluation, and storage of biological specimens for genetic or molecular analysis in this trial/future use {33}

As described above “Additional consent provisions for collection and use of participant data and biological specimens {26b}” section, there will be no biological specimens collected.

### Statistical methods

#### Statistical methods for primary and secondary outcomes {20a}

The primary outcome of this study is the occlusion rate in 7 days. The calculation formula is:

$$\mathrm{Occlusion}\;\mathrm{rate}=\frac{\mathrm{Complete}\;\mathrm{occlusion}+\mathrm{Partial}\;\mathrm{occlusion}}{\mathrm{Number}\;\mathrm{of}\;\mathrm{enrolled}\;\mathrm{cases}}\times100\%$$

A superiority test will be conducted on the occlusion rate of the two groups. The chi-square test will be used to compare the occlusion rates between the two groups. The occlusion rate and 95% confidence interval will be calculated for the intervention group. Simultaneously, the 95% confidence intervals (CIs) for the difference in the occlusion rate between the test group and the control group will be calculated. If the upper limit of the confidence interval is less than the superiority threshold (0), the conclusion of superiority is deemed valid.

Validity analysis will be conducted on both the full analysis set (FAS) and per protocol set (PPS). All baseline demographic data analysis will be conducted on the FAS, and safety evaluations will be based on the safety analysis set. The FAS refers to the set of subjects analysed according to the intention-to-treat (ITT) principle, including all subjects who will be randomized and intervened. For subjects who failed to complete the full efficacy evaluation, non-responder imputation (NRI) will be used to handle the missing data.

With respect to the secondary outcomes, comparisons between the two groups will be made using the group *t* test or Wilcoxon rank-sum test for measurement data according to distribution and the chi-square test or Fisher’s exact probability method for count data. 

#### Interim analysis {21b}

A total of 14 medical institutions will participate in this study, and the progress of the study is expected to be relatively rapid; therefore, no interim analysis is planned.

#### Methods for additional analyses (e.g. subgroup analyses) {20b}

Subgroup analyses are not planned.

#### Methods in analysis to handle protocol non-adherence and any statistical methods to handle missing data {20c}

For possible missing data during the study, the analysis will be carried forward for missing primary outcomes, and the specific carryover method is described in “Statistical methods for primary and secondary outcomes {20a}” section. Catheter occlusion is defined as a partial or complete occlusion of an intravascular catheter, resulting in blocked or restricted infusion of fluid or medication. For subjects who failed to complete the primary outcome evaluation, the method of worst-case imputation will be used to handle missing values. That is, if subjects withdraw early or for any other reason and the primary outcome is missing, the decision of whether or not catheter occlusion occurred will be treated as occlusion.

#### Plans to give access to the full protocol, participant-level data, and statistical code {31c}

The authors plan to publish the protocol, statistical analysis plan, and results. Detailed publication information can be obtained by e-mail to the corresponding author.

### Oversight and monitoring

#### Composition of the coordinating centre and trial steering committee {5d}

The PIs serve as the study supervisor. A project management team was formed consisting of PIs from 14 centres. The group will be responsible for monitoring the progress of the research process and any deviations. The study established an information communication group consisting of PIs from 14 medical institutions, head nurses, SIs, study nurses, and others directly involved in the study. The PI of each centre reports the progress of patient enrolment and adverse events every week. Supervision includes remote supervision and on-site supervision; remote supervision adopts the form of online reporting; and on-site supervision is supervised by the leading unit by arranging SIs to the subcentre for on-site supervision. Surveillance includes access to informed consent, CRF completion, and trial interventions.

#### Composition of the data monitoring committee, its role, and reporting structure {21a}

Because this is not a high-risk study and the study period is short, no specific data monitoring committee (DMC) has been established. However, the research data will be supervised by the scientific research department of the leader centre for a long time.

#### Adverse events reporting and harms {22}

The expected possible complications include catheter occlusion, catheter-related bloodstream infection, thrombosis, and catheter prolapse. All adverse events and unexpected consequences will be evaluated and recorded. The study nurses will monitor the occurrence of complications daily. If an adverse event occurs, the study nurses will report the situation, outcome, and relationship with the trial. In the event of a serious adverse event, the clinical research coordinator will assist the investigator in filling out the “Serious Adverse Event Report Form” as soon as possible, reporting it to the institution’s office and ethics committee, and faxing it to the national State Food and Drug Administration (SFDA), Jiangsu SFDA, and sponsor within 24 h and retaining the fax record.

#### Frequency and plans for auditing trial conduct {23}

The implementation and quality improvement of the interventions will be discussed in weekly group meetings with the study PI, co-investigators, and investigators.

#### Plans for communicating important protocol amendments to relevant parties (e.g. trial participants, ethical committees) {25}

The Medical Ethics Committee of Drum Tower Hospital affiliated to Nanjing University School of Medicine has approved this study, and we will inform it as well as all pertinent ethical committees of the study partners of any protocol amendments and necessary procedure changes. All changes will be noted in the study registration as well. All patients will sign written informed consent before randomization.

#### Dissemination policy {31a}

To share our findings with the scientific community and foster scholarly discourse, we will publish all pertinent study results in scholarly journals. The investigators will also present all the study findings at conferences and gatherings for scientists. Bylines for articles will be ranked based on their actual contributions.

## Discussion

Although PICCs and totally implantable venous access devices (TIVADs) are both better than CVCs in terms of the occlusion rate [26, 27], PICCs and TIVADs are more suitable for long-term infusion therapy patients, while CVCs are more suitable for short-term infusion therapy and surgery patients. A 25–38% occlusion rate was found for the CVC, and a new flushing and locking technique was designed to solve this problem. The conventional catheter flushing technique involves flushing the catheter before and after infusion or under other special circumstances. Considering that the pulsatile flushing technique requires a certain amount of operator skill and that occlusion may occur during drug infusion, we wonder if it is possible to design a technique for continuous flushing so that the catheter is always flushed and whether the drug is being infused, which effectively prevents catheter occlusion. Conventional KVO infusion may result in haemodilution or fluid overload; therefore, we will use an elastic pump with a volume of 50 ml, a flow rate of 2 ml/h, and maintain the infusion pressure at 55 kPa, which is greater than the central venous pressure to replace conventional KVO infusion.

This study offers a completely new way to flush and lock catheters. In this way, the nurse only has to learn to attach and remove the elastic pump, saving time by not having to flush the catheter as often. From a theoretical point of view, continuous flushing might be a good way to avoid catheter occlusion. We hope this study will provide an evidence-based basis for catheter maintenance.

## Trial status

Protocol version 2, July 20, 2022

Started recruitment: February 1, 2023

Protocol submission: March 29, 2023

Recruitment completion: June 2, 2023

Data collection completion: July 31, 2023

### Supplementary Information

Supplementary Material 1. Informed consent form.We used the SPIRIT Checklist to assist with presenting this protocol.

Supplementary Material 2. Checklists.

## Data Availability

The leader unit has access to the final database. Other partner units can access their own database or access other units' data after communication. After the article is published, readers can ask the corresponding author for data upon reasonable request.

## Abbreviations

PIVC: Peripheral intravenous catheter
PICC: Peripherally inserted central catheter
CVC: Central venous catheter
KVO: Keep vein open
CINAS: Catheter Injection and Aspiration
SOP: Standardized operating procedure
CRF: Case report form
CRBSI: Catheter-related bloodstream infection
CRT: Catheter-related thrombosis
PI: Principal investigator
SI: Sub-investigator
