# Supplementary material for: The effect of different flushing and locking techniques on catheter occlusion rates in central venous catheters: protocol for a multicentre, randomized controlled, parallel-group, open-label, superiority clinical trial
Source: Trials. 2024 Jun 12;25:380. doi: 10.1186/s13063-024-08141-6 (PMC11170772; doi:10.1186/s13063-024-08141-6)
Supplement: Supplementary file 1 — Supplementary Material 1. Informed consent form.We used the SPIRIT Checklist to assist with presenting this protocol. [file 13063_2024_8141_MOESM1_ESM.docx]

Supplementary File


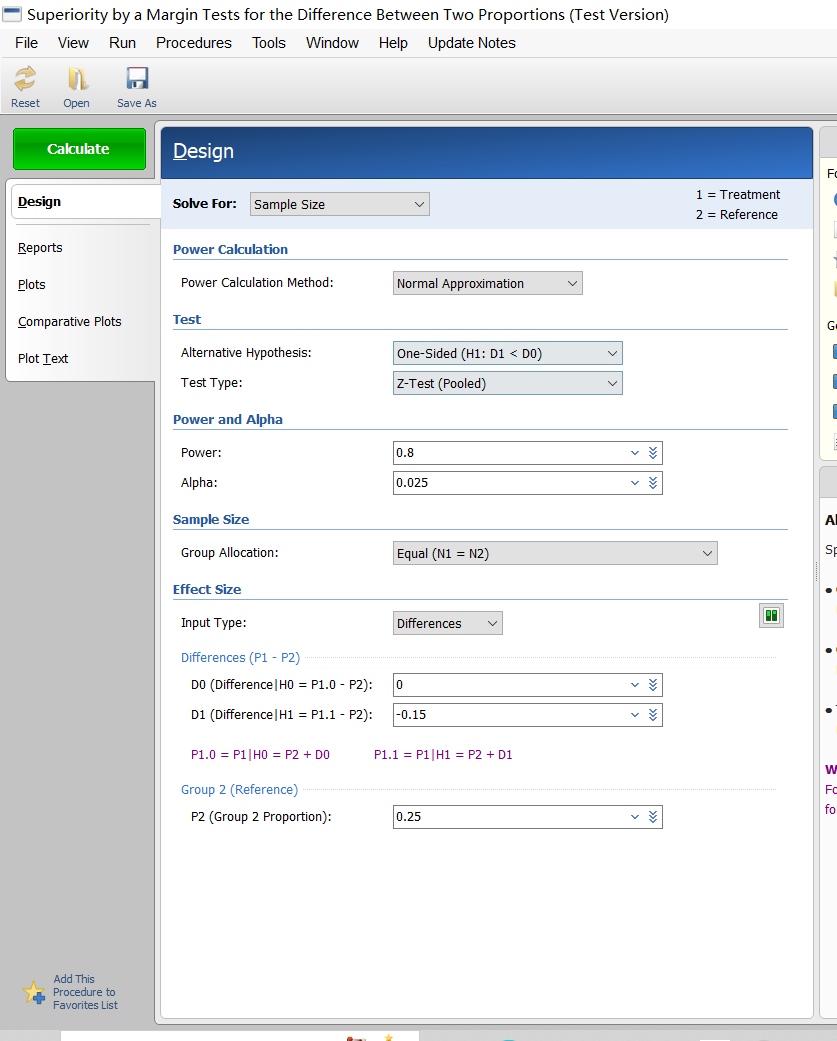


Figure 1.Screenshot of sample size calculation


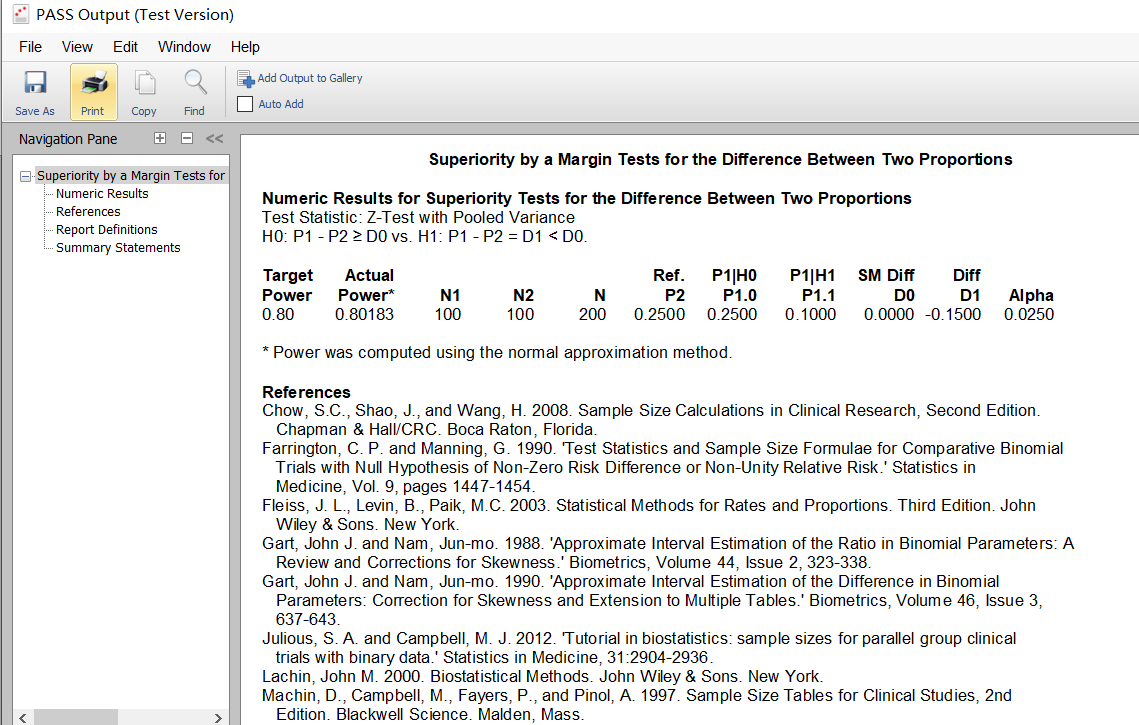


Figure 2.Screenshot of sample size calculation
